# Facilitators and barriers of breastfeeding late preterm infants according to mothers’ experiences

**DOI:** 10.1186/s12887-016-0722-7

**Published:** 2016-11-08

**Authors:** Maria Lorella Giannì, Elena Bezze, Patrizio Sannino, Elena Stori, Laura Plevani, Paola Roggero, Massimo Agosti, Fabio Mosca

**Affiliations:** 1 Fondazione I.R.C.C.S. Ca Granda Ospedale Maggiore Policlinico, Neonatal Intensive Care Unit Department of Clinical Science and Community Health, University of Milan, Via Commenda 12, 20122 Milan, Italy; 2 Fondazione I.R.C.C.S. Ca Granda Ospedale Maggiore Policlinico, S.I.T.R.A. Basic Education Sector, Via Francesco Sforza 28, 20122 Milan, Italy; 3 Neonatologia e Terapia Intensiva Neonatale, Polo Universitario F. Del Ponte, Viale Borri 54, 21100 Varese, Italy

**Keywords:** Late preterm infants, Breastfeeding, Facilitators, Barriers

## Abstract

**Background:**

Late preterm infants account for the majority of preterm births. They are at an increased risk of neonatal mortality and morbidity and are less likely to initiate breastfeeding and to be exclusively breastfed at discharge compared to infants born at term. The aim of this study was to identify the facilitators and barriers to breastfeeding during hospital stays according to the experiences of mothers of late preterm infants.

**Methods:**

We conducted a cross-sectional questionnaire survey. Mothers who intended to breastfeed and had given birth to a newborn admitted to level I and II care, with a gestational age of 34 0/7 to 36 6/7 weeks, were enrolled. Sociodemographic data, neonatal variables, mode of feeding and feeding status at discharge were also collected.

**Results:**

A total of 92 mothers who had given birth to 121 infants were enrolled. At discharge, any human milk was fed to 94 % of infants, with exclusively human milk being fed in 43 % of cases; exclusively formula was fed to 6 % of infants. In the multivariate analysis, having expressed breast milk was independently associated with an increased risk of being fed with either any human milk or formula only (OR = 2.73, 95 % CI 1.05–7.1, *p* = 0.039), whereas being encouraged to practice kangaroo mother care tended to have a protective effect (OR = 0.46, 95 % CI 0.2–1.06, *p* = 0.07).

**Conclusions:**

Based on the present findings, health care professionals should strive to fully implement breastfeeding support for mothers of late preterm infants who intend to breastfeed, in particular optimizing breast milk expression and promoting kangaroo mother care. Further studies are needed to gain further insight into the complex interplay of the factors that modulate breastfeeding outcome in late preterm infants.

## Background

Late preterm infants, classified as infants born from 34 0/7 to 36 6/7 weeks’ gestation, account for nearly three-quarters of preterm births and are at an increased risk of neonatal mortality and morbidity (i.e., jaundice, poor feeding, respiratory distress, hypoglycaemia and sepsis) [1]. In addition, long-term negative health outcomes, such as neurodevelopmental delays, have also been reported [2].

Given the health benefits that breast milk confers to both mothers and infants, breastfeeding is recommended as the normal and unequalled method for feeding infants, including preterm ones [3]. However, breastfeeding late preterm infants is challenging, as emphasized by the fact that mothers that had given birth to late preterm infants are less likely to initiate breastfeeding and to achieve exclusively breastfeeding at discharge compared to mothers that had given birth to infants born at term. In addition, mothers of late preterm infants who do succeed in breastfeeding show a reduced duration of lactation [4, 5].

Although a growing body of evidence exists on the factors that have been identified to be negatively associated with successful breastfeeding in term infants, the factors affecting the feeding of late preterm infants appear to be complex but still relatively understudied [2, 5]. Within this context, a closer examination of breastfeeding outcomes accounting for neonatal factors, such as infants’ health status and the different degrees of their developmental maturity, physiological and psychological maternal factors and social and system factors has been advocated to develop a customized breastfeeding support strategy for the late preterm population [1, 6]. Specifically, of the individual determinants, intention to breastfeed has been demonstrated to be predictive of positive breastfeeding outcomes, provided that adequate support is offered [6].

To our knowledge, there is a paucity of data concerning the breastfeeding experiences of mothers of late preterm infants. Myers et al. [7] performed a survey including 15 mothers who gave birth to infants with a gestational age ranging from 24 to 37 weeks, thus including late preterm infants, who were admitted to a neonatal intensive care unit and identified the facilitators and barriers of breastfeeding in neonatal intensive care units according to the mothers’ experiences. Kair et al. [8] investigated the experience of breastfeeding late preterm infants in a cohort of 44 mothers who were interviewed by phone for the entire time they breastfed, up to a maximum of 12 months after delivery. The authors reported that, according to the interviewed mothers, breastfeeding was a positive bonding experience for them and their infants. However, mothers reported latching difficulties, problems with milk supply and inadequate lactation support after discharge.

The aim of this study was to identify the facilitators and barriers to breastfeeding during hospitalization according to the experience of mothers who intended to breastfeed and gave birth to late preterm infants.

## Methods

### Design and setting

We conducted a cross-sectional questionnaire survey involving mothers who had given birth to late preterm infants who were admitted to the authors’ institution from July to October 2015. Approval from the institutional review board was obtained, and consent was obtained from the mothers concomitantly with the fulfilment of the questionnaires.

### Sample

The inclusion criteria were the following: mothers with a good comprehension of the Italian language who intended to breastfeed and had given birth to a newborn who was admitted to level I and II care, with a gestational age from 34 0/7 to 36 6/7 weeks. The exclusion criteria were mothers who presented contraindications to breastfeeding or who had chosen not to breastfeed and mothers of newborns who were admitted exclusively to level III care and/or had been transferred to another institution.

According to our internal clinical protocol [9], late preterm infants with a birth weight ≥1900 g, regardless of gestational age, were admitted to level I care, provided that their clinical conditions were stable, that is without need for ventilatory and/or cardiovascular support, and that they were able to maintain a stable body temperature and no nutritional support was required. Infants with a birth weight <1900 g and/or requiring any type of nutritional support were admitted/transferred to either level II or III care, according to their clinical conditions.

## Nutritional practices

According to our internal nutritional procedures, all preterm infants with birthweight < = 1900 g, irrespective of gestational age, qualify for parenteral nutrition or intravenous fluids and initiate enteral or oral feeding within the first day of life. Oral feeding is started when gestational age is ≥34 weeks, provided that the infants are in stable clinical conditions, that is showing cardio respiratory stability. All mothers are encouraged to breastfeed their infant, when stable, or to express their milk immediately after birth. Furthermore mothers are supported to learn how to feed their infants according to the cue based feeding method [9, 10]. When human milk is unavailable or insufficient, formula feeding is initiated. On the contrary, infants who present with any clinical condition that could hinder the beginning of enteral/oral nutrition, irrespective of birthweight and/or gestational age, receive exclusively parenteral nutrition/intravenous fluids [11].

### Data collection procedures

Participation in the study was voluntary. The questionnaire required approximately 10–15 min to be completed. The investigator in charge of the study delivered the questionnaires to the mothers the day before their infant was discharged. Returned completed questionnaires from each mother implied their consent to participate in the study. The voluntary nature of the participation and participants’ anonymity were assured throughout the study.

### Instruments

The facilitators and barriers of breastfeeding were evaluated using a modified version of the questionnaire developed by Myer et al. [7] The modified questionnaire was translated into Italian using back-translation. Before starting the study, the Italian version of the modified questionnaire was submitted to a group of mothers to clarify any doubts regarding item comprehension. The Italian version of the questionnaire comprised 14 items, nine investigating the maternal experiences with breastfeeding and five investigating the knowledge and competencies acquired by the mothers during their hospital stay.

The mothers were asked whether they had previous breastfeeding experience, whether they knew women who had breastfed (if the answer was yes, they were invited to specify whether the experience was positive or not) and whether they had attended a pre-pregnancy class (items 1, 2, 3).

Item 4 investigated the mother’s knowledge regarding the benefits of breastfeeding for the mother and the infant (an additional area was provided to allow the mothers to report which benefits they specifically knew), and item 5 addressed maternal perceptions of the breastfeeding support provided by the hospital staff during their hospital stay. Mothers were also asked whether they needed to express breast milk (item 6) and whether their infant was first fed breast milk by bottle (item 10). The mothers who answered yes to item 6 were further asked to indicate when they had first expressed breast milk and the frequency of milk expression. Items 7, 8, and 9 investigated whether the mothers had been taught to recognize the indicators of infants’ hunger and to evaluate infants’ suckling and whether they had been encouraged to practice kangaroo mother care. While items 1 to 10 were dichotomous questions, the mothers were instructed to score items 11 to 14 on a 4-point Likert scale (4 = strongly disagree, 3 = mildly disagree, 2 = mildly agree and 1 = strongly agree).

There were also two additional areas designated for specifying the barriers and facilitators to breastfeeding that the mothers, according to their experiences, had encountered during their hospital stay.

The following maternal variables were collected: age, ethnicity, marital status, education, mode of delivery, parity, singleton or twin pregnancy, spontaneous or assisted pregnancy, and occurrence of any comorbidities (i.e., preeclampsia, diabetes, placenta praevia) during pregnancy. The following neonatal variables were also recorded: gender, birth weight, gestational age, weight at discharge, length of hospital stay, care level of admission, and Apgar score at 1 and 5 min. Gestational age was based on the last menstrual period and the first-trimester ultrasonogram. The infants with a birth weight <10th or ≥10th percentile for gestational age on the basis of Fenton’s growth chart [12] were respectively classified as having a weight that was small for gestational age or appropriate for gestational age. A record was made of the occurrence of any comorbidity such as respiratory distress syndrome, defined as the need for any respiratory support, sepsis, defined as the presence of a positive blood culture, hypoglycaemia, defined as a plasma glucose level <45 mg/dl, and jaundice requiring phototherapy.

The mode of feeding (directly at the breast, exclusively by bottle, or a mixture of both) and the feeding status (exclusively human milk, any human milk or exclusively formula) at discharge were also collected. Infants fed any extent of human milk, irrespective of the quantity or the exclusivity, were categorized as fed any human milk [13].

The maternal and neonatal variables were collected from the medical records after obtaining the consent from the mothers.

### Statistical analysis

The data are presented as the mean ± standard deviation or n (percentage). With regard to the items that were scored on a 4-point Likert scale, for the analysis, the answers were categorized into two groups (agree and disagree).

The associations between the items on the questionnaire (Yes vs No or Agree vs Not Agree) and mode of feeding at discharge (either any human milk or formula fed vs being exclusively fed human milk) were assessed using univariate logistic regression analysis. A multiple logistic regression model, including the items that were significantly associated with mode of feeding at discharge in the univariate analysis, was used to identify the determinants of being exclusively breastfed at discharge according to the mothers’ experience. To avoid collinearity between items 6 and 10, only item 6 was entered in the model.

Statistical analyses were performed using SPSS (Statistical Package for the Social Sciences) version 12 software (SPSS Inc., Chicago, IL, USA).

## Results

During the study period, 104 mothers were screened for eligibility, and 92 fulfilled the inclusion criteria of the study, for a total of 121 infants. All the mothers that fulfilled the inclusion criteria agreed to participate to the study. The basic characteristics of the mothers and their infants are shown in Table 1. Most of the mothers enrolled in this study were Caucasian and married, with an average age of 35 ± 0.16 years and an education level less than or equal to 13 years. More than half were primiparous and underwent a caesarean section after a pregnancy that was characterized by the occurrence of comorbidity in 39 % of the cases. Sixty-two mothers gave birth to a single child, whereas 30 mothers gave birth to twins; however, only 59 of the 60 twins were enrolled because one mother had one of her twins admitted to another institution. Half of the twin pregnancies followed the induction of pregnancy. A total of 95 % of the enrolled mothers achieved breastfeeding; among those breastfeeding, 51 % exclusively breastfed. Overall, the enrolled infants had a mean gestational age at birth of 35.2 ± 0.84 weeks, with a mean birth weight of 2430 ± 414 g (range 1445–3560 g). Sixty percent of infants were admitted to level I care, whereas 40 % of infants were either admitted or transferred to level II care according to their clinical conditions. A total of 24 infants (20 %) were admitted to level III care and were then transferred to level II care when their clinical conditions improved. Eighty-six infants (71 %) developed at least one comorbidity during their hospital stay; specifically, the most frequent comorbidities were respiratory distress, hypoglycaemia and jaundice requiring phototherapy, which occurred in 23, 18 and 35 % of infants, respectively. Thirty-three infants were affected by more than one comorbidity.

**Table 1:** Basic characteristics of the enrolled mother-infant pairs

Mothers (*n* = 92)	Mean ± SD
Age (years)	35 ± 0.16
	*N* (%)
Marital status	
Married	58 (63)
In a relationship with infant’s father but not married	28 (30)
Single parent	6 (7)
Ethnicity	
Caucasian	74 (80)
Hispanic American	8 (9)
African	6 (7)
Asian	4 (4)
Maternal education level	
≤ 13 years	62 (67)
> 13 years	30 (33)
Caesarean section	54 (59)
Occurrence of comorbidity during pregnancy	36 (39)
Assisted pregnancy	17 (18)
Primiparous	62 (67)
Infant (*n* = 121)	Mean ± SD
Gestational age at birth (weeks)	35.2 ± 0.84
Birth weight (g)	2430 ± 414
Apgar 1’	8.5 ± 0.89
Apgar 5’	9.6 ± 0.69
Length of hospital stay (days)	9.9 ± 6.5
Weight at discharge (g)	2374 ± 363
	*N* (%)
Twins	59 (49)
Males	69 (57)
Small for gestational age infants	18 (15)

At discharge, full oral feeding was achieved exclusively at breast by 20 % of infants and exclusively by bottle by 9 % of infants; 71 % of infants achieved full oral feeding being fed partially by bottle and partially at breast. At discharge, any human milk was fed to 94 % of infants, with exclusively human milk being fed in 43 % of cases and exclusively formula being fed to 6 % of infants. Table 2 reports the responses to the questions investigating the maternal experiences with breastfeeding.

**Table 2:** Answers to the questions investigating maternal experiences with breastfeeding

Item *n*		Yes	No
1	Have you had previous experience breastfeeding?	29 (32)	63 (68)
2	Do you know women who have previously breastfed?	80 (87)	12 (13)
3	Did you attend a pregnancy course?	35 (38)	57 (62)
4	Do you know the benefits of breastfeeding for you and your child?	61 (66)	31 (34)
5	Did you feel you were adequately supported by the breastfeeding consultant and/or by the health care providers during your hospital stay?	85 (92)	7 (8)
		Disagree	Agree
11	I am satisfied with my experience breastfeeding during my hospital stay	18 (20)	78 (80)
12	I feel comfortable communicating with health care professionals about breastfeeding	9 (10)	83 (90)
13	I feel that the hospital staff is supportive of breastfeeding/pumping breast milk	2 (2)	90 (98)
14	I feel that there are enough resources available to assist me with breastfeeding	34 (37)	58 (63)

Most of the mothers who answered the questionnaire reported that they did not have previous experience of breastfeeding and that they knew other women who had breastfed, 51 % of whom reported a positive experience with breastfeeding. Although more than half of the enrolled mothers stated that they had not attended a pregnancy class, 66 % declared that they were aware of the benefits associated with breastfeeding both for the mother and for the baby. Specifically, according to their knowledge, they indicated the following: protection from infections (95 %), positive effect on infant-mother bonding (38 %), nutritional benefits (25 %), enhancement of infant’s development (15 %), helpful for mothers in the postpartum period (11 %), protection from development of cancer in the mother (10 %), protection against allergies (7 %), low cost (7 %), protection against obesity development (5 %), pregnancy spacing (5 %), and protection against sudden infant death syndrome (2 %).

Based on the average scores of their responses, the mothers felt adequately supported with regard to breastfeeding during their hospital stay, and, accordingly, they were satisfied overall with their experience with breastfeeding and felt comfortable communicating with health care professionals about breastfeeding.

In Table 3, the items regarding the knowledge and competencies acquired by the mothers during their hospital stay are reported. Most mothers needed to express breast milk and first fed their baby by bottle. Breast milk was first expressed after 24 h in 60 % of the mothers, within the first 24 h in 27 %, within the first 12 h in 9 % and within 6–8 h from delivery in 4 % of the mothers. With regard to the frequency of milk expression, the majority of the mothers expressed breast milk four to five times per day, whereas only 4 % expressed breast milk at least eight times per day. While most of the mothers stated that they had been taught to recognize signs of hunger in their infant, only half of them were taught to evaluate how the infant suckled at their breast and were encouraged to practice kangaroo mother care.

**Table 3:** Answers to the questions investigating the knowledge and competencies acquired by the mothers during their hospital stay

Item *n*		Yes	No
6	Did you need to express breast milk either manually or with an electronic device?	68 (74)	24 (26)
7	Were you taught to recognize signs of hunger in your infant?	80 (87)	12 (13)
8	Were you taught to evaluate how the infant suckled your breast?	52 (56)	40 (44)
9	Have you been encouraged to practice kangaroo mother care?	51 (55)	41 (45)
10	Was your infant first fed breast milk by bottle?	69 (75)	23 (25)

In the univariate analysis, of the items on the questionnaire, only having expressed breast milk (item 6) and having first fed the baby by bottle (item 10) were independently associated with an increased risk of being fed with either any human milk or with formula only (OR = 3.08, 95 % CI 1.2–7.8, *p* = 0.018 and OR = 3.28, 95 % CI 1.2–8.5, *p* = 0.02, respectively). Having been encouraged to practice kangaroo mother care (item 9) was protective (OR = 0.40, 95 % CI 0.2–0.9, *p* = 0.03). In the multivariate analysis, only having expressed breast milk remained independently associated with an increased risk of being fed with either any human milk or with formula only (OR = 2.73, 95 % CI 1.05–7.1, *p* = 0.039), although having been encouraged to practice kangaroo mother care tended to remain protective (OR = 0.46, 95 % CI 0.2–1.06, *p* = 0.07).

With regard to the open-ended questions, the main points that were indicated by the mothers as facilitating and inhibiting breastfeeding are reported in Table 4.

**Table 4:** Items/intervention facilitating and inhibiting breastfeeding according to mothers’ experiences during hospital stay

Items/Intervention Facilitating Breastfeeding	Percent
Being taught how to position the infant at the breast	32
Availability of a breast pump	31
Availability of expert lactation support	30
Having the baby in the same room as the mother or being able to see him/her without any time constraints	27
Breastfeeding support group	13
Previous positive experience breastfeeding	12
Kangaroo mother care	2
Availability of written information about the importance of breastfeeding	2
Items/Intervention Inhibiting Breastfeeding	
Inadequate suckling capacity due to prematurity	26
Separation from infant	25
Presence of medical devices such as phototherapy	21
Maternal concerns about providing an adequate milk supply	20
Infant’s drowsiness	18
Presence of comorbidity	17
Having twins	9
Insufficient support from expert lactation consultant	7
Maternal perception of being inadequate	6
Maternal stress due to infant’s clinical conditions	6

Regarding the interventions identified as facilitating breastfeeding by the mothers, in most cases, mothers stressed the importance of expert lactation support, including education by health care professionals. The factors that were reported more frequently by mothers as inhibitors of breastfeeding were related to either prematurity or situations that are commonly associated with preterm birth such as infant’s drowsiness, occurrence of comorbidities, presence of medical devices, separation from infant and having twins.

## Discussion

The results of the present study highlighted the main facilitators and barriers to breastfeeding according to late preterm infants’ mothers. The majority of mothers felt they were adequately supported by the breastfeeding consultant and/or by the health care providers during their hospital stay, stated they were satisfied with their experience of breastfeeding and felt comfortable in communicating with health care professionals about breastfeeding. Accordingly, the percentage of infants who were breastfed at discharge in the present study was 94 %, which is higher than previous data published in the literature [2, 4, 5]. This finding could be explained by the fact that, according to the inclusion criteria of the study, all enrolled mothers intended to breastfeed. Indeed, intention has been found to be predictive of the initiation and continuation of breastfeeding, provided adequate support is offered [6]. Furthermore, in our sample, the majority of mothers, although not having attended a prenatal course, were aware of the beneficial effects of breastfeeding on health outcomes. The perception of the benefits of breastfeeding has actually been described as one of the major reasons for the initiation and continuation of breastfeeding in mothers of preterm infants because it is regarded as a means of compensating for the early birth [14].

Despite the potential beneficial effects of breastfeeding in the late preterm population, late preterm infants are at a higher risk of lower initiation of breastfeeding than term infants [2, 4, 5]. Breastfeeding initiation in the U.S. has been reported by Radtke et al. [1] to range around 59–70 %. Demirci et al. [5] reported a lower breastfeeding prevalence in late preterm than in term infants, despite a positive trend from 54 % in 2003 to 61.8 % in 2009. Rayfield et al. [4] reported that 85.6 % of late preterm infants received any human milk at 10 days; this timing can be considered equivalent to the discharge in our study because the mean hospitalization length was 9.9 days. However, the percentage of infants fed exclusively human milk at discharge in the present study was 43 %, which is relatively low in comparison to the data reported in the literature. Ayton et al. [2] reported that 59.7 % of late preterm infants were exclusively breastfed at discharge. However, it must be considered that the hospital in their study was a WHO/UNICEF Baby Friendly accredited hospital, and hence, the study’s setting could be characterised by a particularly strong support for breastfeeding. Wooldridge et al. [15] found an exclusive breastfeeding rate of 60 % in moderately preterm infants, born at 30 to 35 weeks' gestation. However, it must be noted that their study also included infants at a gestational age younger than 34, who have been reported to present better breastfeeding outcomes than late preterm infants, probably due to the higher breastfeeding support and extra care placed on infants requiring intensive care [16]. The relatively low exclusive breastfeeding rate found in our study may be partially explained by the fact that the majority of the mothers delivered by caesarean section and were primiparous, known risk factors for breastfeeding failure [2]. Comorbidities, such as pregnancy-induced hypertension, were present in 39 % of cases and could have delayed lactogenesis II and prevented breastfeeding establishment. In addition, 71 % of infants presented at least one comorbidity, which may have led to the mother and child being separated [1]. Indeed, in the present study, the possibility of having the baby in the same room as the mother or being able to see him without time constraints was reported to be effective in promoting breastfeeding in 27 % of cases. A total of 24 (20 %) infants had to be admitted to level III care until their clinical conditions stabilized, which could have further prolonged the separation from their mother and increased maternal anxiety; this, in turn, could have negatively affected lactogenesis II [1, 17]. Indeed, NICU-admitted infants have been reported to be particularly susceptible to poor breastfeeding outcomes [18]. Additionally, maternal stress due to infants’ clinical condition has been identified by the interviewed mothers as a barrier to breastfeeding, although in a relatively low number of cases.

Although the majority of mothers in the present study felt that they were adequately supported in breastfeeding, 37 % of them reported that they felt that there were not enough resources available to assist them, stressing the need for increased lactation support. The association between breastfeeding support and breastfeeding outcome in 579 late preterm infants has been investigated by Rayfield et al. [4] The authors found that mothers who were not adequately helped with breastfeeding during their hospital stay were more likely not to breastfeed at 10 days compared to mothers who received enough help. Consistent with these findings, in the present study, the availability of expert lactation support and of a breastfeeding support group was reported by mothers as facilitating breastfeeding in 30 % and 13 % of cases, respectively.

Factors related to the infant, with special regard to the varying degrees of infant developmental immaturity, could have further complicated the feeding situation. In fact, preterm infants have been reported to present latching difficulty, lethargy, ineffective suckling and metabolic disturbances that could further predispose them to poor breastfeeding outcomes and inadequate emptying of the breast, which would then require mothers to express breast milk [1, 19]. Consistent with these data, in the present study, the mothers stated that the most frequent barriers to breastfeeding were ineffective suckling, infants’ drowsiness, presence of a medical device and separation from their infant. However, it has to be taken into consideration that very preterm infants have been shown to develop competent nutritive sucking at low gestational ages. Specifically, Nyqvist KH [20] investigated breastfeeding behavior in 15 preterm infants, born at gestational ages between 26 and 31 weeks, and reported that they initiated breastfeeding from a postmenstrual age of 29 weeks, reaching full breastfeeding at a median age of 35 weeks of gestational age.

Having twins was also reported by the mothers to be a barrier to breastfeeding. However, the results concerning the effect of preterm plural births on breastfeeding are inconsistent [21, 22].

In contrast, the availability of a breast pump was identified as an intervention facilitating breastfeeding in 31 % of cases. However, in the binary logistic regression, having expressed breast milk in addition to having first fed the baby by bottle was independently associated with an increased risk of being fed with either any human milk or with formula vs being exclusively breastfed. These findings could be partially explained by the fact that although the majority of mothers felt that the hospital staff was supportive of breastfeeding/expressing breast milk, only 4 % of mothers expressed breast milk within the first 6–8 h of delivery. Furthermore, the frequency of breast milk pumping was only higher than eight times a day in 4 % of mothers, whereas the majority of them expressed milk four to five times per day. Previous studies have demonstrated that at 3 weeks after delivery, women who pump within an hour of birth produce higher volumes of milk than those who begin at 6 h after birth [23]. In addition, high pumping, that is a minimum of 6.25 times a day, has been identified as a significant predictor of later milk supply [24]. Furthermore, although the majority of the mothers in the present study were taught to recognize signs of hunger in their infants, only one out of two reported being shown how to evaluate infants’ suckling behaviour. The importance of teaching parents to observe and interpret their infant’s behaviour is widely acknowledged [25, 26]. Specifically, with regard to feeding, “cue-based” oral feeding, which is the ability to recognize signs of readiness for feeding and signs of distress that occur during feeding, has been reported to enhance the development of preterm infants’ oral skills, allowing the infant to learn to feed efficaciously and safely [9, 10, 20, 27]. Accordingly, being taught how to place the infant at one’s breast was reported to be an intervention that facilitated breastfeeding in 32 % of cases. Having been encouraged to practice kangaroo mother care showed a tendency of being protective towards being fed any human milk and/or formula vs being exclusively breastfed. The importance of skin-to-skin contact in promoting breastfeeding outcomes has been emphasized by Moore et al. [28], who reported that healthy newborns were more likely (risk ratio = 1.27; 95 % CI = 1.06 to 1.53) to be breastfed at 1 to 4 months after birth if they had received skin-to-skin contact at birth or soon after delivery. Furthermore, kangaroo mother care has been reported to be effective in the establishment and in promoting long-term breastfeeding in preterm infants [29, 30].

Indeed, during kangaroo mother care infant has unrestricted access to the mother's breasts. It is widely acknowledged that the milk is effectively eliminated from the breast via the milk ejection reflex, triggered by infant sucking, and, as a result, milk synthesis is increased in response to breast emptying. In addition, it has been demonstrated that emptying of the mammary gland does not occur following a synchronous pattern due to different timing of myoepithelial cell response [31].

The present study, although clinically interesting and addressing a relatively large number of mothers, has some limitations. First, the enrolled mothers were interviewed at a single institution; hence, the results of the present study may not apply to all hospitals. Second, it investigated the experience of mothers who gave birth to late preterm infants who were admitted both to level I and II care, which could actually reflect very different situations both in terms of clinical conditions and modalities of breastfeeding support. Furthermore, no record of the duration of mother-infant separation, when it occurred, was made.

## Conclusions

Based on the present findings, health care professionals should exert efforts to fully implement breastfeeding support for mothers of late preterm infants who intend to breastfeed, in particular optimizing breast milk expression and promoting kangaroo mother care. Further studies are needed to gain further insight into the complex interplay of the factors modulating breastfeeding outcomes in late preterm infants.

## Abbreviations

AGA: Adequate for gestational age
CI: Confidence interval
GA: Gestational age
OR: Odds ratio
SD: Standard deviation
SGA: Small for gestational age
